# Exploring the molecular mechanism of comorbidity of autism spectrum disorder and inflammatory bowel disease by combining multiple data sets

**DOI:** 10.1186/s12967-023-04218-z

**Published:** 2023-06-08

**Authors:** Jinyi Zhu, Haoran Meng, Li Zhang, Yan Li

**Affiliations:** 1 School of Clinical Medicine, Affiliated Hospital of Weifang Medical University, Weifang Medical University, Weifang, China; 2 Key Laboratory of Birth Regulation and Control Technology of National Health Commission of China, Shandong Provincial Maternal and Child Health Care Hospital affiliated to Qingdao University, Jinan, 250014 China

**Keywords:** Autism spectrum disorder (ASD), Inflammatory bowel disease (IBD), WGCNA, Bioinformatics, DEGs, Hub genes

## Abstract

**Background:**

Autism spectrum disorder (ASD) is a neurodevelopmental disorder that is difficult to diagnose. Inflammatory bowel disease (IBD) is a common chronic digestive disease. Previous studies have shown a potential correlation between ASD and IBD, but the pathophysiological mechanism remains unclear. The purpose of this research was to examine the biological mechanisms underlying the differentially expressed genes (DEGs) of ASD and IBD using bioinformatics tools.

**Methods:**

Limma software was used to evaluate the DEGs between ASD and IBD. The GSE3365, GSE18123, and GSE150115 microarray data sets were acquired from the Gene Expression Omnibus (GEO) database. We then performed 6 analyses, namely, Gene Ontology (GO) and Kyoto Encyclopedia of Genes and Genomes (KEGG) functional annotation; weighted gene coexpression network analysis; correlation analysis of hub genes with autophagy, ferroptosis and immunity; transcriptional regulation analysis of hub genes; single-cell sequencing analysis; and potential therapeutic drug prediction.

**Results:**

A total of 505 DEGs associated with ASD and 616 DEGs associated with IBD were identified, and 7 genes overlapped between these sets. GO and KEGG analyses revealed several pathways enriched in both diseases. A total of 98 common genes related to ASD and IBD were identified by weighted gene coexpression network analysis (WGCNA), and 4 hub genes were obtained by intersection with the 7 intersecting DEGs, which were PDGFC, CA2, GUCY1B3 and SDPR. We also found that 4 hub genes in the two diseases were related to autophagy, ferroptosis or immune factors. In addition, motif–TF annotation analysis showed that cisbp__M0080 was the most relevant motif. We also used the Connectivity Map (CMap) database to identify 4 potential therapeutic agents.

**Conclusion:**

This research reveals the shared pathogenesis of ASD and IBD. In the future, these common hub genes may provide new targets for further mechanistic research as well as new therapies for patients with ASD and IBD.

**Supplementary Information:**

The online version contains supplementary material available at 10.1186/s12967-023-04218-z.

## Introduction

Autism spectrum disease (ASD) is a neurodevelopmental disease that is characterized by restricted and repetitive patterns of behaviour and interest, as well as a lack of social communication and engagement. As reported by the U.S. Centers for Disease Control in 2021, the overall prevalence of ASD among 8-year-olds in the United St`ates as of 2018 was 1 in 44, and the prevalence of ASD among boys was 4.2 times higher than that of girls [1]. The overall prevalence of ASD in Asian children remains unclear due to the current lack of ASD surveillance networks in Asia. IBD is a chronic disorder that is linked to immune system issues, changes in gut microbiota, vitamin malabsorption, and anaemia. It is defined by chronic inflammation of the gastrointestinal tract, including the two main subtypes of Crohn’s disease and ulcerative colitis. The prevalence of IBD varies significantly by country, with higher rates in Europe (CD: 322/100,000 in Germany; UC: 505/100,000 in Norway) and North America (CD: 319/100,000 in Canada; UC: 286/100,000 in the United States) [2]. However, consistent increases in recent decades have been reported in South America, Africa, and Asia [2]. More research has revealed that people with ASD often have immunological problems and related gastrointestinal symptoms, with gastrointestinal symptoms being the most prevalent comorbidity. A study of 48,762 American children with ASD and 243,810 healthy controls found that the children with ASD had a 47% and 94% higher risk of developing Crohn’s disease (CD) and ulcerative colitis (UC), respectively [3].

Although there is evidence that IBD and ASD may be related, the precise mechanism explaining the coexistence of the two conditions is yet unknown. The putative gut–brain connection has frequently been invoked to explain the potential connection between ASD and IBD. Children with ASD exhibit aberrant intestinal permeability and altered intestinal microbiomes, according to previous studies [4, 5]. Furthermore, it has been suggested that genetics may contribute to the association between ASD and IBD [6]. IBD may also be associated with perinatal factors associated with ASD, such as anaemia, vitamin malabsorption and perinatal immunological abnormalities [7]. According to studies in mice, maternal immune activation (MIA) causes the offspring to display an autism-like phenotype as well as become immunologically sensitive and more vulnerable to bacteria-caused intestinal inflammation [6]. MIA can cause an increase in maternal IL-17 A, which in turn affects the chromatin accessibility of offspring CD4 + T cells by altering the maternal intestinal flora, enhancing the inflammatory phenotype, and thus causing an immunological sensitized phenotype [8].

With the advancement of bioinformatics and the use of gene chips in recent years, bioinformatics analysis has grown to be an indispensable part of the biomedical field. Common transcriptional patterns could offer fresh perspectives on the shared aetiology of ASD and IBD. For this investigation, we obtained three microarray data sets from the GEO database (GSE3365, GSE18123, and GSE150115). The research used extensive bioinformatics and enrichment analysis for the purpose of identifying the genes that are expressed differently in ASD and IBD, as well as their functions. To connect modules with one another and with external clinical features, the weighted gene coexpression network analysis (WGCNA) method was used. Based on this, we built a regulatory network comprising the hub gene and immune/autophagy/ferroptosis genes and examined the hub gene expression profile in single cells from IBD patients. The final steps included a study of the transcriptional regulation of hub genes, the prediction of related miRNAs using the miRcode database, and identification of suggested medications or chemicals using the CMap database. The further study of the abovementioned hub genes shared between ASD and IBD is expected to shed light on the biological mechanisms underlying these two diseases.

## Materials and methods

### Data download

The Series Matrix File data file of GSE18123 was downloaded from the NCBI GEO public database [9]. GPL570 is the licence for the annotation platform. With 33 cases in the normal group and 31 instances in the ASD group, a total of 64 sets of transcriptome data were included. The GSE3365 Series Matrix File data file was downloaded, and the annotation file is licenced under GPL96. A total of 127 transcriptome data sets from 42 normal cases and 85 IBD cases were included. The limma package [10] was used to compare the normal and disease-affected groups, and the screening criteria for DEGs were P value < 0.05 and |logFC|>0.585. The GSE150115 data file, with a total of 5 samples, was downloaded for single-cell correlation analysis.

### Analysis of GO and KEGG functions

The Metascape database (www.metascape.org) [11] was used for annotation and visualization, and the DEGs were analysed by Gene Ontology (GO) and Kyoto Genome Encyclopedia (KEGG) [12] pathway analysis to obtain the biological functions and signalling pathways involved in disease occurrence and development. Enrichment was deemed statistically significant when the overlap was ≥ 3 and p ≤ 0.01.

### WGCNA for coexpression network construction

Weighted gene co-expression network analysis (WGCNA) was performed to identify coexpressed gene modules and investigate the relationship between the gene network and phenotype, as well as the core genes in the network. The WGCNA-R package [13] was used to build the coexpression network of all genes in the data set, and this algorithm was used to identify the top 10,000 genes with variance for further analysis. To estimate the network connection, the weighted adjacency matrix is converted into a topological overlap matrix (TOM), and its clustering tree structure is constructed using a hierarchical clustering method. The branches of the clustering tree represent different gene modules, and the colours represent the various modules. Genes are categorized according to their expression patterns, genes with comparable functions are grouped into one module, and tens of thousands of genes are separated into several modules by gene expression patterns according to the genes’ weighted correlation coefficient.

### Regulatory network analysis of hub genes

The R package “RcisTarget” [14] was used in this study to predict transcription factors. RcisTarget bases all of its calculations on the presence of certain motifs. A motif’s normalized enrichment score (NES) is determined by the total number of motifs in the database. In addition to the motifs labelled in the source data, we also inferred additional annotations based on motif similarity and gene sequence. To determine the overexpression of each motif across a gene set, the area under the curve (AUC) for each motif–motif set pair was first determined based on the recovery curve calculation of the gene set versus motif ordering. The AUC distribution of all motifs in the gene set was used to calculate the NES of each motif. We utilized rcitarget.hg19.motifdb.cisbpont.500 bp as the gene-motif rankings database.

### miRNA analysis

Small noncoding RNAs known as microRNAs (miRNAs) have been proven to regulate gene expression by either promoting mRNA degradation or inhibiting mRNA translation. Therefore, we further analysed whether some miRNAs in hub genes regulate the transcription or degradation of some dangerous genes. We obtained miRNAs related to hub genes through the miRcode database [15] and visualized the miRNA network of genes through Cytoscape software.

### Single-cell sequencing analysis

The data were first processed using the Seurat package [16], and then tSNE method analysis as used to identify the spatial links between each pair of clusters; the clusters were then annotated using the celldex package, and some cells that are crucial to the development of the were are annotated. Finally, using the logfc.threshold option of FindAllMarkers, we extracted the marker genes for each cell subtype from the single-cell expression profile. Genes with |avg_log2FC| > 1 and p_val_adj < 0.05 were chosen as separate marker genes for each cell subtype.

### Potential therapeutic drugs associated with hub genes

A promising tool for drug screening is the Connectivity Map (CMap; https://clue.io/) database, which can forecast molecularly targeted drugs based on DEGs [17]. Based on cell expression profile data processed with 164 drugs/small molecule compounds and overexpression or gene knockout tools, the database explores the network of drugs/small molecule compounds, genes, and disease states with the L1000 analysis platform. In this study, we predicted possible chemical drugs for the treatment of ASD and IBD using gene expression profiling and the CMap database. The results showed that interference with the functions of specific genes has a potential therapeutic effect because the expression pattern of the corresponding gene is diametrically opposed to the specific expression pattern of the disease. This negative score represents the expression pattern of the corresponding gene.

### Statistical analysis

Statistical analysis was carried out using R language (version 4.0), and p < 0.05 was considered to indicate statistical significance.

## Results

### Screening of DEGs from GSE3365 and GSE18123

**Fig. 1:**
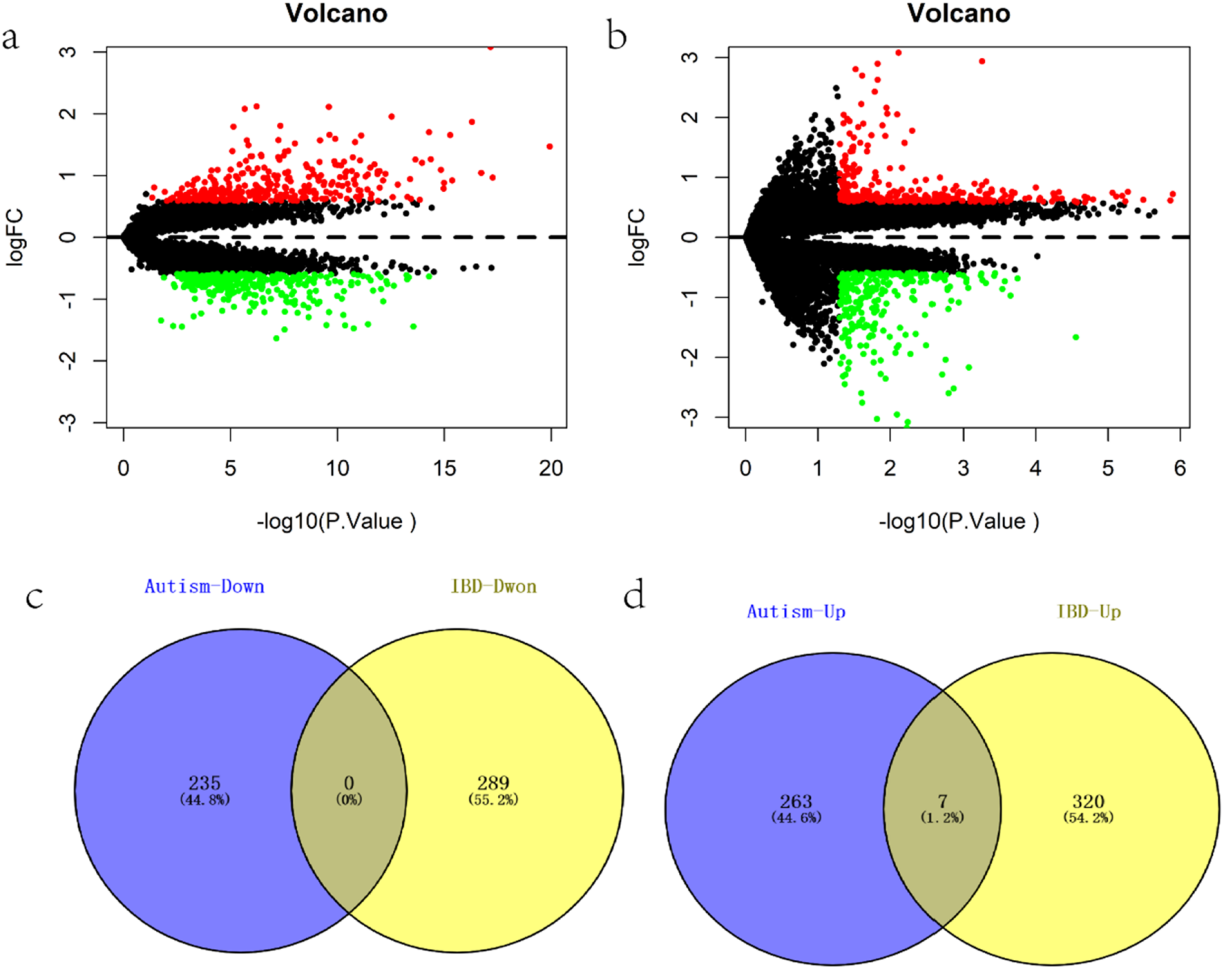
Identification of common differential genes. **a** Volcano map of DEGs in IBD, green indicates down-regulated DEGs, and red indicates up-regulated DEGs. **b** Volcano map of DEGs in ASD, green indicates down-regulated DEGs, and red indicates up-regulated DEGs. **c**, **d** Venn diagram identifies co-upregulated and co-downregulated DEGs

Using data from the open NCBI GEO database, we obtained the Series Matrix File data file for GSE3365, which addresses inflammatory bowel diseases. A total of 127 sets of transcriptome data, including 42 from normal samples and 85 from IBD samples, were included in the annotated file GPL96. The DEGs between the two groups of samples were identified using the limma package. P value < 0.05 and |logFC| > 0.585 were the criteria for definition of a DEG, and a total of 616 DEGs (including 327 upregulated and 289 downregulated genes) were identified (Fig. 1a). Then, the GSE18123 Series Matrix File data file relating to ASD was downloaded. The substrate for annotations was GPL 570. Altogether, 64 sets of transcriptome data were used, 33 of which were from normal samples and 31 were from ASD samples. To calculate the difference between the two groups, the limma program was used. A total of 505 DEGs, including 270 upregulated and 235 downregulated genes, were screened under the following conditions: P value < 0.05 and |logFC|>0.585 (Fig. 1b). Then, we intersected the up- and downregulated DEGs of the two data sets and obtained a total of 7 intersecting DEGs (Fig. 1c and d).

### Functional enrichment analysis

**Fig. 2:**
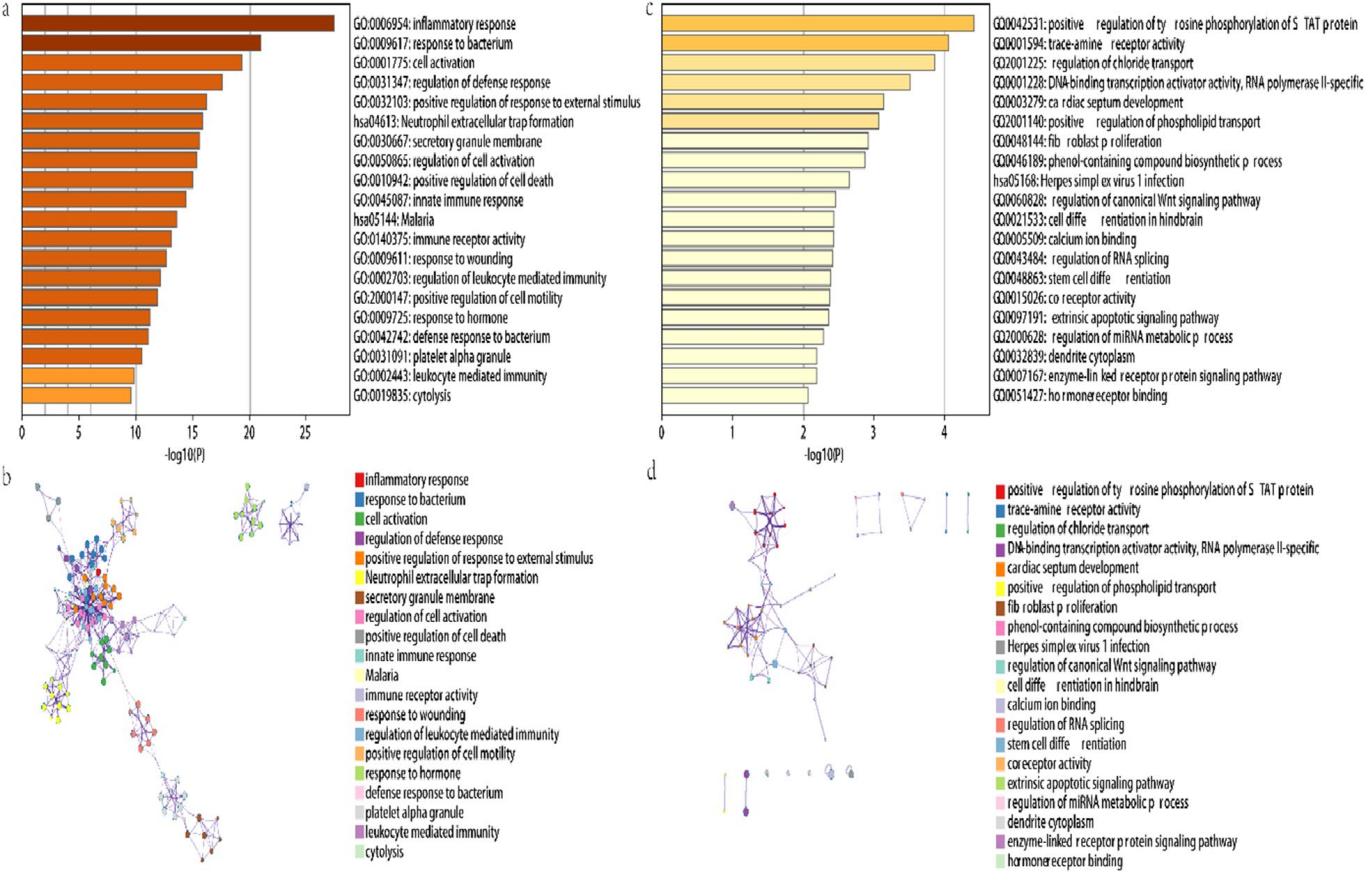
Enrichment analysis. GO and KEGG enrichment analysis of DEGs based on the Metascape database. A cluster network of enrichment pathways; nodes that share the same cluster are usually close to each other. **a**, **b** represent IBD, and **c**, **d** represent ASD

We conducted pathway analysis on the DEGs from these two data sets. The findings revealed that GSE3365’s 616 intersecting DEGs were primarily enriched in pathways such as inflammatory response, response to bacteria, and cell activation (Fig. 2a); the 505 DEGs of GSE18123 were mainly enriched in positive regulation of tyrosine phosphorylation of STAT protein, trace-amine receptor activity, regulation of chloride transport and other pathways (Fig. 2b).

### Coexpression network construction and hub module identification

**Fig. 3:**
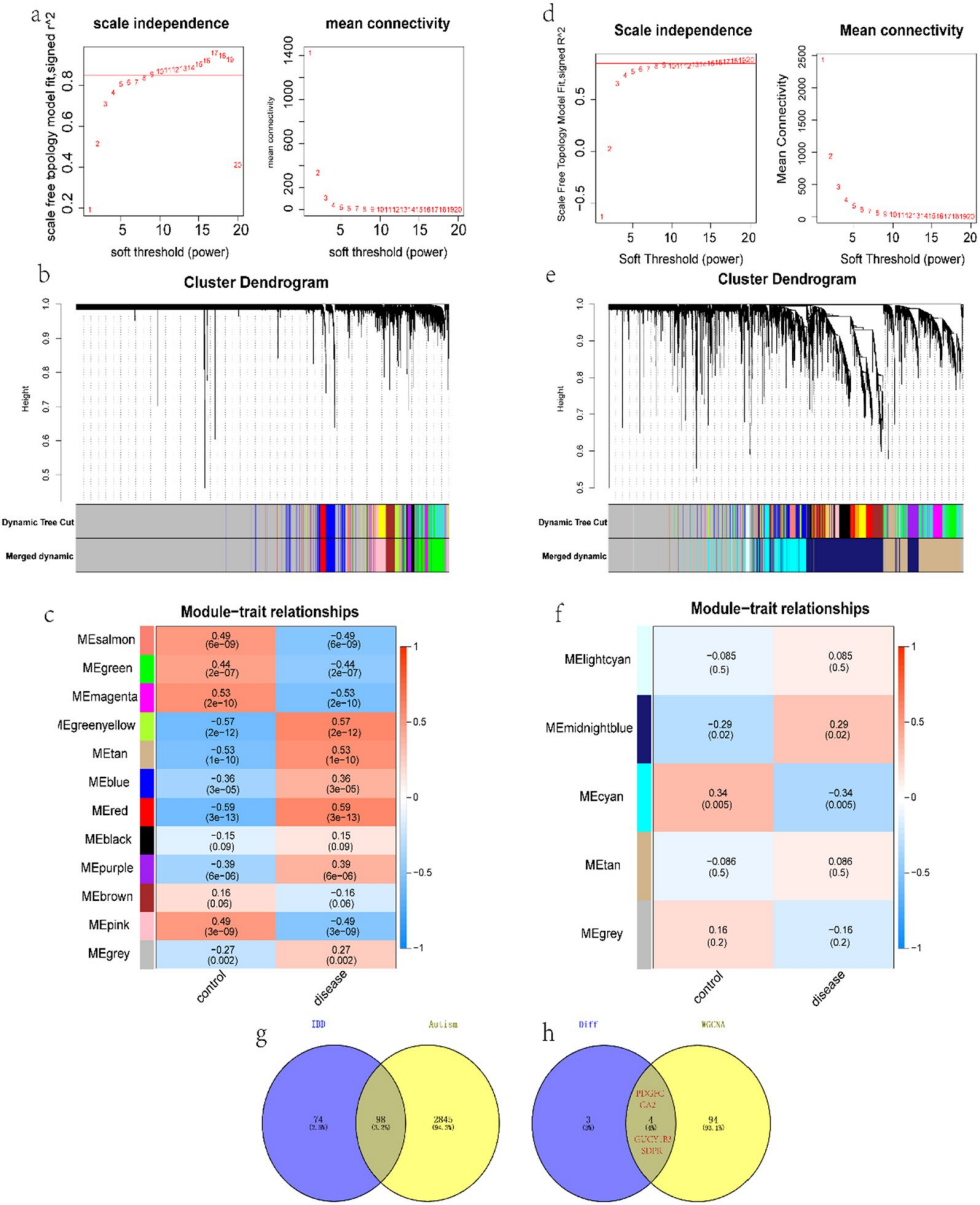
WGCNA. **a** Scaleless index and average connectivity of individual soft thresholds for IBD. **b** Dendrogram of IBD gene clustering, with different colours representing different modules. **c** Heatmap of the correlation between module characteristic genes and IBD; blue indicates negative correlation, and red indicates positive correlation. **d** Scaleless index and average connectivity of each soft threshold for ASD. **e** Dendrogram of gene clustering of ASD, with different colours representing different modules. **f** Heatmap of the correlation between module characteristic genes and ASD; blue indicates negative correlation, and red indicates positive correlation. **g**, **h** Venn diagrams identify module intersection genes and hub genes

From the expression profile data of GSE3365, the WGCNA network was further constructed to explore the related coexpression network in IBD (Additional file 1). The threshold β was set to 15, and then the tom matrix was used to identify the gene modules. A total of 12 gene modules were found, namely, black (166), blue (527), brown (365), green (743), green yellow (113), grey (7031), magenta (132), pink (435), purple (128), red (172), salmon (78), and tan (110), among which the red module had the strongest inverse relationship with the disease (cor = 0.59, p=(3e − 13)) (Fig. 3a and c). Next, we constructed a WGCNA coexpression network for the expression spectrum of GSE18123 (autism). The threshold β was set to 15, and the tom matrix was used to identify the gene modules. Five gene modules in total were found, namely, cyan (1273), grey (3645), light cyan (82), midnight blue (2943), and tan (2057), among which the midnight blue module had the strongest inverse relationship to disease (cor = 0.29, p=(0.02)) (Fig. 3d and f). Subsequently, the red module of GSE3365 was intersected with the midnight blue module of GSE18123, and 98 intersecting genes were obtained (Fig. 3g). We then compared this set of genes with the 7 ASD/IBD intersecting DEGs identified above, and a total of 4 intersection genes were obtained (Fig. 3h): PDGFC, CA2, GUCY1B3, and SDPR. These four genes were considered the hub genes for our follow-up studies.

### Relationship between hub genes and genes related to key regulatory mechanisms

**Fig. 4:**
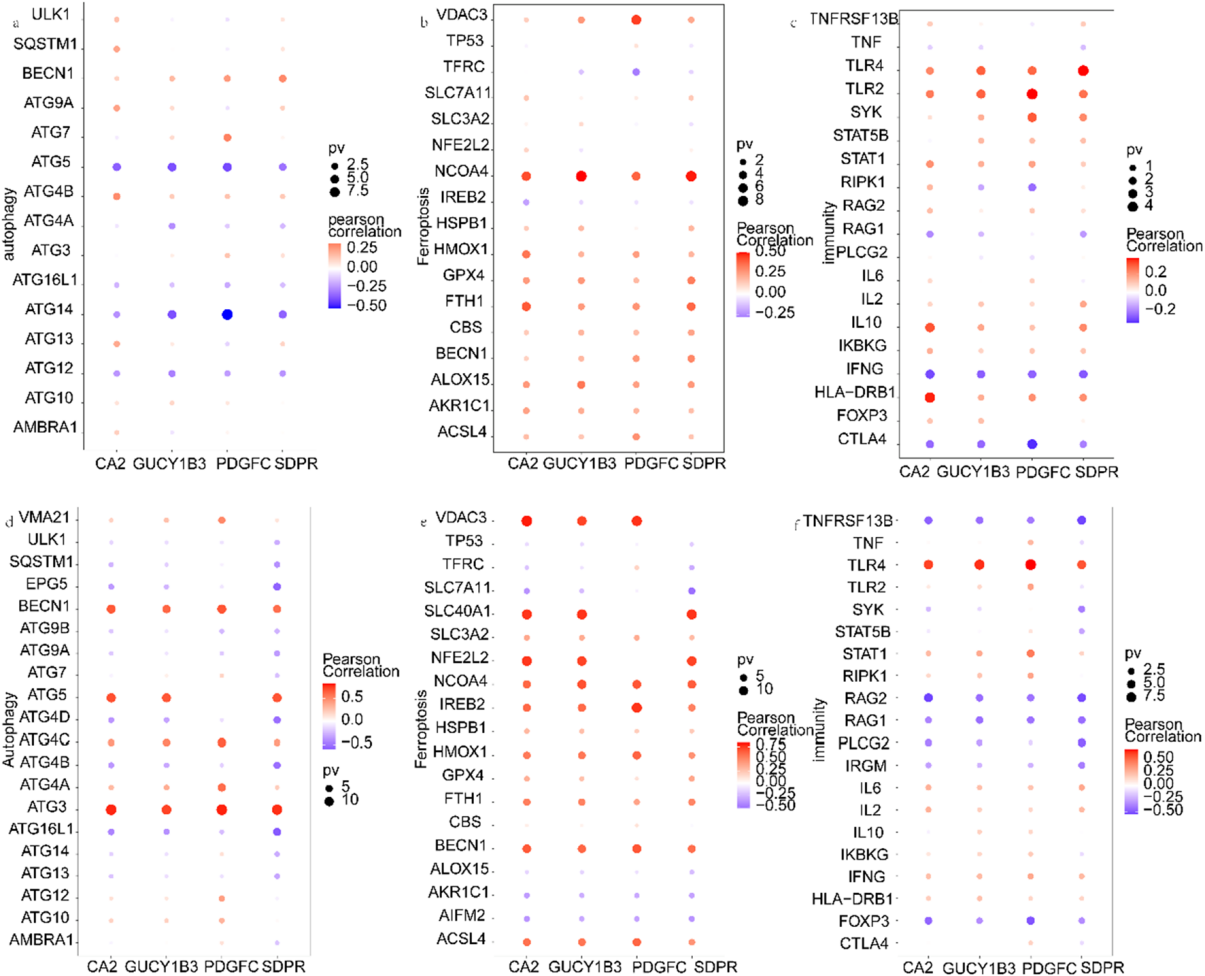
Relationship of hub genes to other genes. **a–c** Correlation of hub genes in IBD with autophagy, ferroptosis, and immune factors. **d**–**f** Correlation of hub genes in ASD with autophagy, ferroptosis, and immune factors

**Fig. 5:**
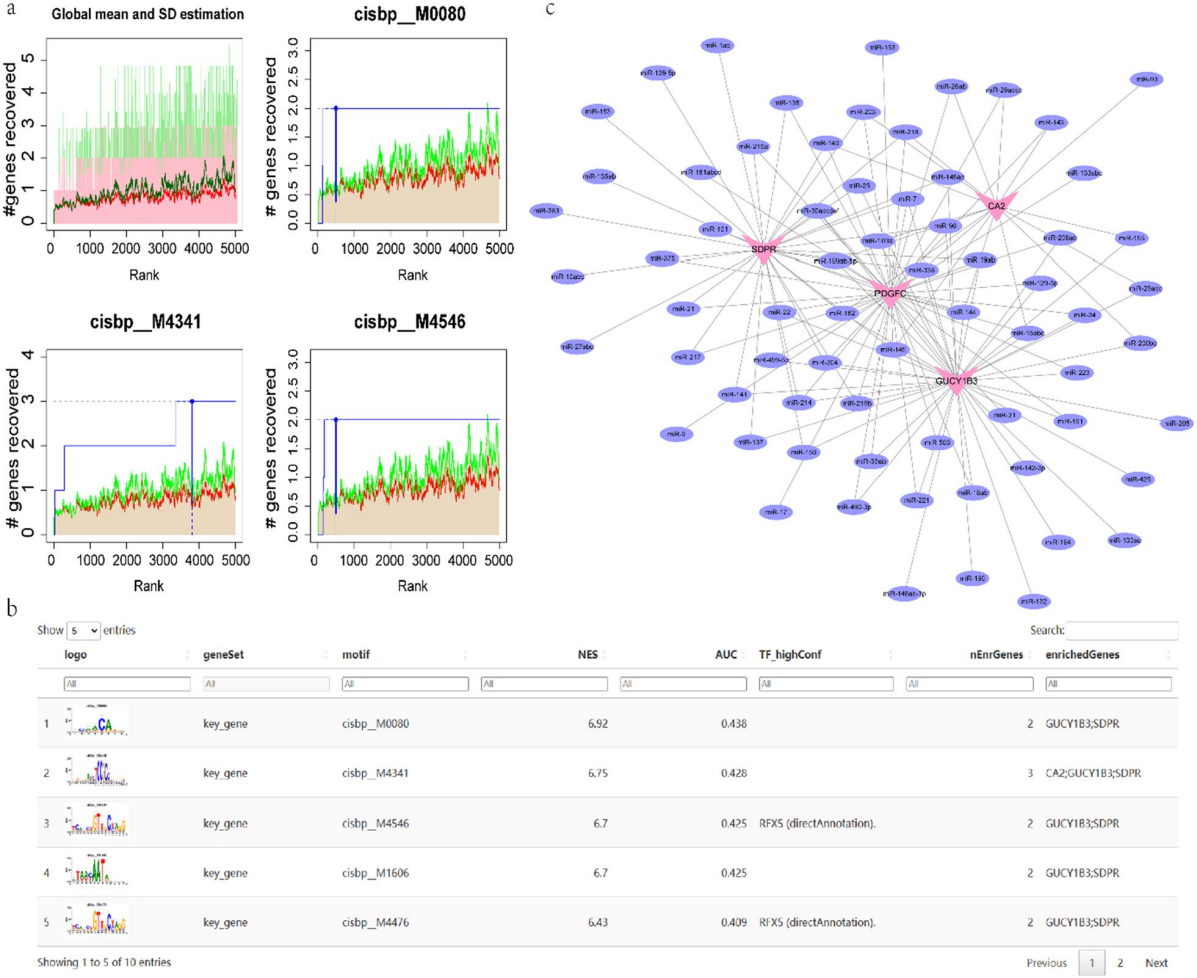
Motif transcriptional regulation analysis. **a** The three motifs with the highest AUC values. The red line is the average of the recovery curve of each motif, the green line is the mean + standard deviation, and the blue line is the recovery curve of the current motif. The maximum distance point (mean + sd) between the current motif and the green curve is the maximum enrichment level selected. **b** Demonstration of the highest motif enrichment of AUC, including NES (standardized enrichment score for motifs in gene sets), AUC (area under the curve), and TF_highConf (transcription factors annotated to motifs). **c** miRNA networks of hub genes, pink for mRNA and purple for miRNA

Then, we obtained the immunity, autophagy and ferroptosis disease gene sets from the GeneCards database (https://www.genecards.org/) and extracted the expression levels of the top 20 genes and the relevance score of the 4 hub genes (Additional file 3, 4, 5, 6, 7, 8). Correlation analysis was then carried out (Fig. 4). We discovered that the 4 hub genes are all controlled by the same transcription factors and other shared regulatory processes. Therefore, the cumulative recovery curve (Fig. 5a), motif–TF annotation, and the selection analysis results of significant genes were subjected to enrichment analysis for these transcription factors. The results revealed that the motif with the highest normalized enrichment score (NES: 6.92) was cisbp__M0080 (Additional file 2). This motif was more prevalent in the GUCY1B3 and SDPR genes. We present all hub gene-enriched motifs and associated transcription factors in Fig. 5b.

### Transcriptional regulation analysis and potential drug prediction

**Fig. 6:**
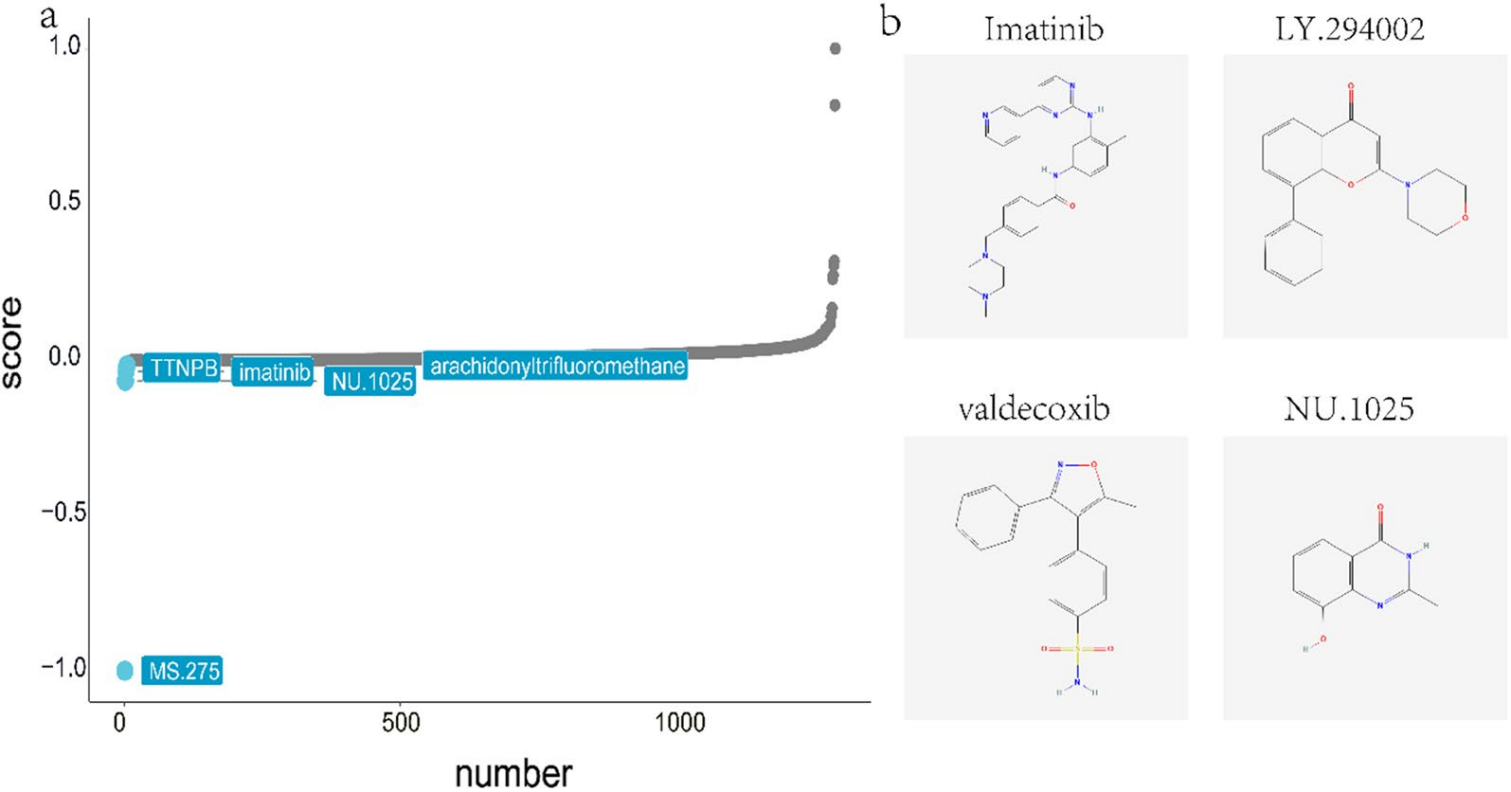
Prediction of potential therapeutic agents for ASD and IBD. **a** Potential therapeutics predicted by the connectivity map database. **b** Molecular structure diagram of imatinib, LY.294002, valdecoxib and NU.1025

We also reverse-predicted 4 hub genes using the miRcode database, obtained 68 miRNAs, and visualized 132 mRNA‒miRNA relationship pairs using Cytoscape (Fig. 5c). Furthermore, we downloaded the drug data from CMap and predicted potential therapeutic drugs using the PharmacoGx package and the 505 intersecting DEGs. The outcomes demonstrated that the expression patterns perturbed by imatinib, LY.294,002, STOCK1N.35,696, PHA.00816795, and X16.phenyltetranorprostaglandin.E2 drug were most negatively correlated with the expression profiles perturbed by disease. This suggests that these drugs could mitigate or even reverse this disease state (Fig. 6a). We present the molecular structures of some of the predicted drugs in Fig. 6b.

### Overview of hub gene expression in single cells

**Fig. 7:**
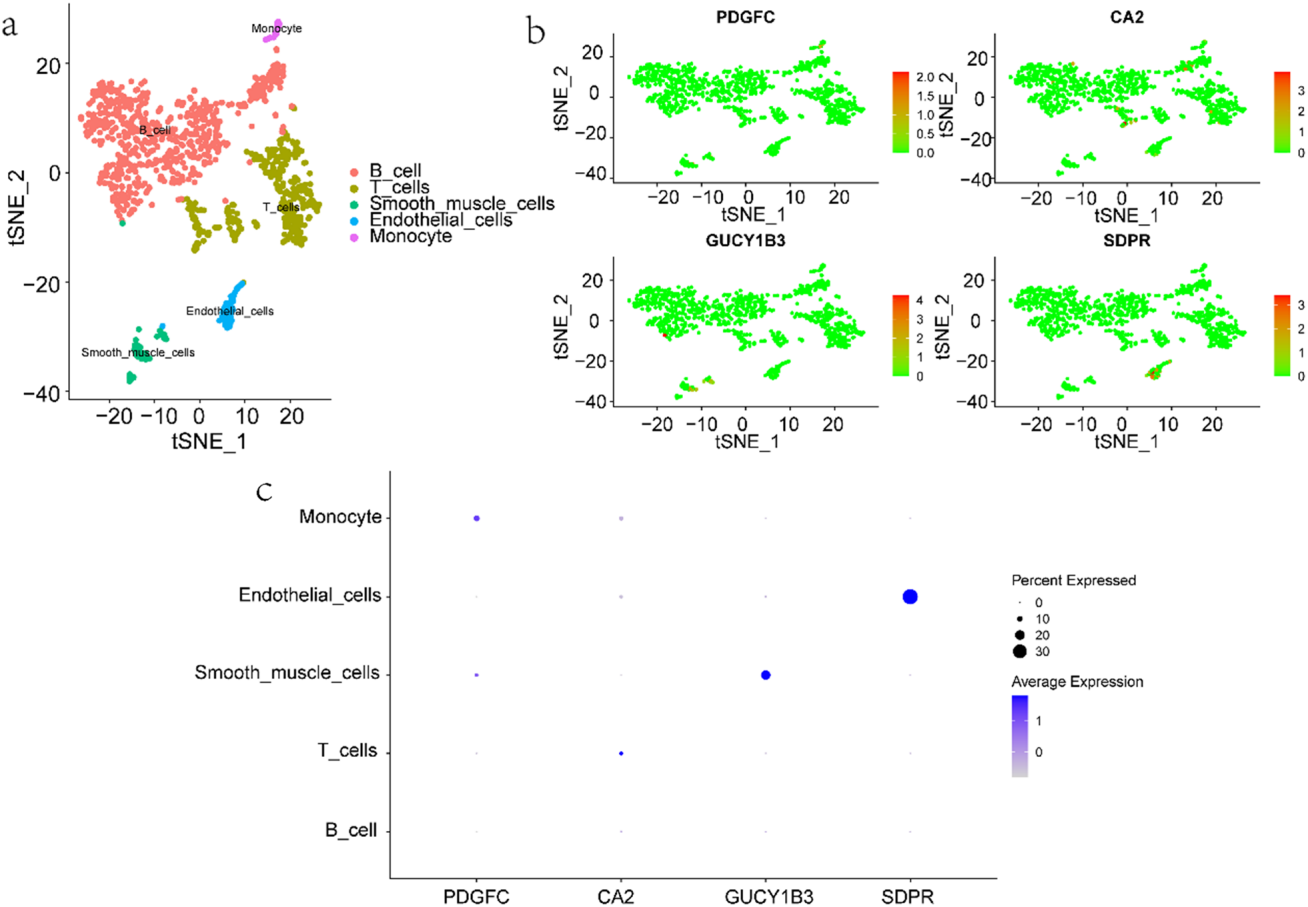
Expression profiles of hub genes in single cells. **a** Cellular subtypes of ulcerative colitis. **b**, **c** Scatter plots and bubble plot of the expression of the 4 hub genes

We downloaded the single-cell data in GSE150115 and performed single-cell analysis through the Seurat package, clustered the cells with the tSNE algorithm, used HumanPrimaryCellAtlasData as the annotation data, and annotated each cluster through the R package SingleR. All cells were annotated into five categories: B_cell, T_cells, Smooth_muscle_cells, Endothelial_cells and Monocytes (Fig. 7a). The expression levels of PDGFC, CA2, GUCY1B3, and SDPR in the five types of cells are shown in Fig. 7b and c.

## Discussion

At present, an increasing number of studies are confirming the link between ASD and IBD. Previous research has demonstrated that IBD is a cooccurring illness that is frequently diagnosed in children with ASD [18]. Children with ASD frequently have gastrointestinal abnormalities and related symptoms, but it is unclear how common these disorders are and how best to treat them [19]. In addition, studies have shown that children with ASD have a higher prevalence of IBD than children without ASD [3, 20]. Walker SJ et al. showed that the molecular profile of the gastrointestinal mucosa in children with ASD overlaps significantly with that of known IBD but has unique features that further support the presence of variants associated with ASD or the prodromal phase of classic IBD [21]. Until now, it has not yet been completely determined how ASD and IBD are related. Consequently, studies of the comorbid mechanism of ASD and IBD have important clinical significance for early recognition and intervention in the disease.

In this study, 3 microarray data sets from ASD and IBD were subjected to various bioinformatics methods, and the GEO database was used to identify 7 DEGs common to ASD and IBD. The 616 DEGs of IBD were primarily enriched in inflammatory response and response to bacterial pathways, according to GO and KEGG analyses. The 505 DEGs of ASD were primarily enriched in positive regulation of tyrosine phosphorylation of STAT protein and trace-amine receptor activity. ASD patients have been found to have immune abnormalities such as imbalanced cytokine response, differences in the number and distribution of immune cells, and neuroinflammation, which were also consistently found in IBD patients [22, 23]. Numerous studies have shown that immunological inflammatory mechanisms are necessary for the onset and development of ASD [24–27], that immune dysfunction is crucial for the pathogenesis of IBD [28], and that the occurrence of immune responses can further fuel intestinal inflammation [29]. Our results suggest a possible course of action for the investigation of the mechanisms underlying the comorbidity of ASD and IBD.

In addition, by constructing a WGCNA coexpression network, 5 ASD-related gene modules and 12 IBD-related gene modules were identified. After intersecting the red module in IBD and the midnight blue module in ASD, we obtained 98 genes, mainly enriched in the platelet alpha granule, blood coagulation, neutrophil extracellular trap formation and other pathways. Then, after intersecting the obtained WGCNA intersection genes with the 7 DEGs, we obtained a total of 4 hub genes for in-depth analysis. WGCNA focuses on the connection between clinical features and coexpression modules in comparison to other bioinformatics approaches, which makes the study results more thorough, more reliable, and more biologically significant [30]. Genes within the same module are believed to be functionally interconnected. This methodology can therefore be used to forecast biologically significant modules and hub genes for biomarkers that may be shared by ASD and IBD.

To understand the underlying mechanisms of autophagy, ferroptosis, and immune factors and the two diseases, we performed correlation analysis of 4 hub genes with information from the GeneCards database. As shown in Fig. 4, the expression of hub genes in IBD and ASD had different degrees of correlation with autophagy, ferroptosis and immune factors. Among them, the ferroptosis-related gene NCOA4 and the immune-related gene TLR4 (toll-like receptor 4) were significantly positively correlated with hub genes in both diseases. Previous studies have shown that intestinal hepato-like tumours are more common in the context of IBD and that hepato-like tumours have recurrent changes in molecular characteristics, including NCOA4-RET fusion [31]. Hughes HK et al. confirmed that TLR4 activation can induce the expression of immune genes and thus affect gene expression in monocytes in children with ASD [32]. In addition, increased intestinal barrier permeability in ASD patients may be triggered by activating the lipopolysaccharide-mediated TLR4-MyD88 (myeloid differentiation factor 88)–NF-κB (nuclear factor kappa B) pathway [33]. At the same time, there is growing evidence that immune system dysfunction, particularly TLR4 signalling pathway dysfunction, plays a key role in the pathogenesis of IBD [34, 35] and that the development of IBD and the benefits of its treatment are both influenced by the TLR4 signalling pathway [36]. These studies suggest that genes related to immunity and ferroptosis-related genes may be a significant factor in the copathogenesis of ASD and IBD.

Transcription factors contribute significantly to regulating gene expression. In this paper, we used RcisTarget to identify significant binding motifs and corresponding transcription factors for hub genes. Based on the extraction of motif sequences, we can predict potential binding sites [37], which helps us further explore possible molecular mechanisms. Small noncoding RNAs called miRNAs (21–25 nucleotides long) can complement the 3′ UTR of target mRNAs and thereby cause mRNA destruction or inhibiting mRNA translation [38]. We constructed an mRNA‒miRNA regulatory network in which miR144, miR145, miR-182, and miR-199ab-5p had the highest average connectivity among the four hub genes. According to research by Lin Z et al., treating inflammatory illnesses by controlling the expression of the miR-144/451 gene and the activation of DCs in IBD patients may be a unique strategy [39]. Other studies have shown significant downregulation of miR-145 in chronic ulcerative colitis compared with normal colonic mucosa [40]. Additionally, Sabaie H et al. identified ANP32A-IT1/hsa-miR-182-5p/S100A2 and RBM26-AS1/hsa-miR-182-5p/S100A2 as two putative DElncRNA–miRNA–DEmRNA axes in the pathophysiology of ASD [41]. Studies on the pathogenesis of ASD have demonstrated that miRNAs directly contribute to the development of ASD [42, 43]. Studies involving suppression of the translation of hub mRNAs involved in neurodevelopment and function in various ASD models have demonstrated that miRNAs play a crucial role in the onset and development of ASD [44, 45]. In this study, the mRNA‒miRNA regulatory network of the hub genes was constructed to identify various miRNAs that may have an impact on ASD and IBD. This study also predicted potential therapeutic drugs for 4 hub genes, which will support future studies of the treatment of the disease.

Finally, a single-cell data set from IBD samples was downloaded for single-cell annotation analysis. Single-cell analysis can be used to detect cellular heterogeneity and elucidate the underlying mechanisms [46]. The annotated cell types were mainly B cells, T cells, smooth muscle cells, endothelial cells and monocytes, in which 4 hub genes were expressed to varying degrees. Frede A et al. have shown that expansion of B cells during intestinal injury impairs epithelial–stromal cell interactions that are required for mucosal healing, which has implications for the treatment of IBD [47]. Maintaining the properties of Foxp3(+) regulatory T cells (Tregs) has been found to be essential for controlling the immune response in the gut [48, 49], and the imbalance between Tregs and T effector cells has been linked to inflammatory bowel disease [50]. Immune cells include B cells, T cells, monocytes and so on. Mitsialis V et al. also used single-cell techniques to identify immune cell populations specific to mucosal and blood samples in IBD patients [51]. The above analysis shows that immunity does play an important role in IBD, consistent with the previous analysis. This provides a reliable direction for the in-depth study of the mechanism of ASD and IBD comorbidity in the future.

Although hub genes associated with ASD [52] and IBD [53] have been analysed previously, few studies have used bioinformatics to explore the copathogenesis and shared influencing factors between the two diseases. According to earlier research, children with ASD are more likely than neurotypical children to acquire IBD [3], and ASD is significantly associated with subsequent IBD [54]. Therefore, this study explores and identifies the common DEGs and hub genes for the first time and analyses the possible potential regulatory factors, which is conducive to further studying the molecular mechanisms of ASD and IBD. However, our study had some limitations. First, our study identified only 4 hub genes. Second, the detailed molecular mechanisms of how the hub genes, miRNAs, and transcription factors impact these diseases are lacking. Third, the function of the hub genes and the in vivo efficacy of the identified potential therapeutic drugs need to be verified by further experimental and clinical research.

## Conclusion

We evaluated transcriptome data from ASD and IBD patients, identified DEGs and hub genes common to both diseases, and performed subsequent analyses such as functional enrichment, WGCNA, transcriptional regulation, and single-cell sequencing. We discovered that there may be copathogenesis between ASD and IBD, which may be mediated by the hub genes. This study will aid in the investigation of the molecular mechanisms underlying ASD and IBD.

All in all, this study provides a possible diagnosis and evaluation method for children with ASD in the future. By exploring the combination of ASD and IBD, we can consider using methods such as detecting immune factors or inflammatory factors to screen ASD in the future, and we can also think about using genetic testing to make a definite diagnosis. In addition, in the future, we can try to conduct precise targeted therapy for children with ASD complicated with IBD through the gene loci screened in this paper. However, the above theories still needs to be further confirmed by clinical experiments.

## Supplementary Information

**Additional file 1: Supplementary method**. Supplementary method provide detailed descriptions of WGCNA parameters settings.

**Additional file 2: Table S1**. Transcriptional regulation analysis of key genes.

**Additional file 3: Table S2**. Pearson association of hub genes with ferroptosis genes in dataset GSE29691.

**Additional file 4: Table S3**. Pearson association of hub genes with ferroptosis genes in dataset GSE3365.

**Additional file 5: Table S4**. Pearson association of hub genes with autophagy genes in dataset GSE29691.

**Additional file 6: Table S5**. Pearson association of hub genes with autophagy genes in dataset GSE3365.

**Additional file 7: Table S6**. Pearson association of hub genes with immunity genes in dataset GSE29691.

**Additional file 8: Table S7**. Pearson association of hub genes with immunity genes in dataset GSE3365.

## Abbreviations

ASD: Autism spectrum disorder
AUC: Area under the curve
CMap: Connectivity map
CD: Crohn’s disease
DEGs: Differentially expressed genes
DElncRNA: Differentially expressed lncRNA
DEmRNA: Differentially expressed mRNA
GEO: Gene expression omnibus
GO: Gene ontology
IBD: Inflammatory bowel disease
KEGG: Kyoto encyclopedia of genes and genomes
MIA: Maternal immune activation
MiRNAs: microRNAs
MyD88: Myeloid differentiation factor 88
NES: Normalized enrichment score
NF-Κb: Nuclear factor kappa B
TOM: Topological overlap matrix
TLR4: Toll-like receptor 4
UC: Ulcerative colitis
WGCNA: Weighted gene co-expression network analysis
