# Supplementary material for: Exploring the molecular mechanism of comorbidity of autism spectrum disorder and inflammatory bowel disease by combining multiple data sets
Source: J Transl Med. 2023 Jun 8;21:372. doi: 10.1186/s12967-023-04218-z (PMC10249282; doi:10.1186/s12967-023-04218-z)
Supplement: Supplementary file 1 — Additional file 1: Supplementary method. Supplementary method provide detailed descriptions of WGCNA parameters settings. [file 12967_2023_4218_MOESM1_ESM.docx]

**GSE3365 and GSE29691-WGCNA parameters**

Screen variance of the first 10000 genes with this algorithm for screening, threshold set to 15, module minimum gene number set to 50, through mergeCloseModules function merge similar modules, merge module clustering tree height set to 0.25.
